# Differential risks of psoriatic arthritis development in patients with varied psoriasis manifestations: a sex- and ethnicity-specific analysis

**DOI:** 10.3389/fmed.2024.1385491

**Published:** 2024-06-21

**Authors:** Bernard Gershater, Katja Bieber, Artem Vorobyev, Marlene A. Ludwig, Henner Zirpel, David A. De Luca, Diamant Thaci, Khalaf Kridin, Ralf J. Ludwig

**Affiliations:** ^1^Lübeck Institute of Experimental Dermatology, University of Lübeck, Lübeck, Germany; ^2^Department of Dermatology, University Hospital Schleswig-Holstein Lübeck, Lübeck, Germany; ^3^Independent Researcher, Groß Grönau, Germany; ^4^Institute and Comprehensive Centre for Inflammation Medicine, University-Hospital Schleswig-Holstein, Lübeck, Germany; ^5^Azrieli Faculty of Medicine, Bar-Ilan University, Safed, Israel; ^6^Unit of Dermatology and Skin Research Laboratory, Barch Padeh Medical Center, Poriya, Israel

**Keywords:** psoriasis, arthritis, risk, pustular psoriasis, pustulosis palmoplantaris, psoriatic arthritis, TriNetX, cohort study

## Abstract

**Objectives:**

This study investigated psoriatic arthritis (PsA) risk across varied psoriasis manifestations, considering sex and ethnicity.

**Methods:**

Using TriNetX, a federated database encompassing over 120 million electronic health records (EHRs), we performed global retrospective cohort studies. Psoriasis vulgaris (Pso), pustulosis palmoplantaris (PPP), and generalized pustular psoriasis (GPP) cohorts were retrieved using ICD-10 codes. Propensity score matching, incorporating age, sex, and ethnicity, was employed. An alternative propensity matching model additionally included established PsA risk factors.

**Results:**

We retrieved data from 486 (Black or African American-stratified, GPP) to 35,281 (Pso) EHRs from the US Collaborative Network. Significant PsA risk variations emerged: Pso carried the highest risk [hazard ratio (HR) 87.7, confidence interval (CI) 63.4–121.1, *p* < 0.001], followed by GPP (HR 26.8, CI 6.5–110.1, *p* < 0.0001), and PPP (HR 15.3, CI 7.9–29.5, *p* < 0.0001). Moreover, we identified significant sex- and ethnicity-specific disparities in PsA development. For instance, compared to male Pso patients, female Pso patients had an elevated PsA risk (HR 1.1, CI 1.1–1.2, *p* = 0.002). Furthermore, White Pso patients had a higher likelihood of developing PsA compared to their Black or African American counterparts (HR 1.3, CI 1.04–1.7, *p* = 0.0244). We validated key findings using alternative propensity matching strategies and independent databases.

**Conclusion:**

This study delineates nuanced PsA risk profiles across psoriasis forms, highlighting the pivotal roles of sex and ethnicity. Integrating these factors into PsA risk assessments enables tailored monitoring and interventions, potentially impacting psoriasis patient care quality.

## 1 Introduction

Psoriasis is a common chronic inflammatory disease, predominately affecting the skin. Psoriatic disease most commonly manifests as psoriasis vulgaris (Pso), which affects 1–3% of the worldwide population (1). Less common psoriasis types are pustulosis palmoplantaris (PPP) and generalized pustular psoriasis (GPP). Compared to Pso, PPP has a lower prevalence of 0.6 to 8.9 cases per 10,000 people (2), while GPP is considered an orphan disease with a reported prevalence ranging from 0.02 to 1.4 cases per 10,000 people (3). Despite significant advancements in the treatment of psoriasis, this disease still imposes a major medical burden (4).

One major contributor to the disease burden of Pso is psoriatic arthritis (PsA). PsA manifests in 8.3 to 30% of Pso patients (5–7), but is most likely underdiagnosed (8, 9). In a prospective study, the risk of PsA amounted to 10.1% in 464 Pso patients during an 8-year follow-up (10). Hence, the risk of PsA in Pso is well-established. However, the reported associations between the two diseases vary greatly, there is only scant data from case–control studies, and with few exceptions (11), the impact of sex and ethnicity remains largely understudied (11, 12). Similarly to Pso, PPP is associated with a significant comorbidity, which predominantly includes PsA. The reported risk of comorbid PsA in PPP is lower when compared to Pso, with 6.31 cases per 1,000 patients as opposed to 11.49 cases per 1,000 patients (13). In a small-scale observational study, 14% of PPP patients were also diagnosed with PsA (14). Collectively, PsA coincides with PPP in 8.6 to 26% of cases (15). Thus, there is a substantial body of evidence supporting an association of PPP with PsA. However, no cohort studies have been performed to determine the risk of PsA in PPP, and data regarding sex- and ethnic-specific risks or controlling for the bias potentially imposed by PsA risk factors, is missing. In GPP, a recent study from Japan highlighted a highly significant association with PsA in GPP patients (16). In this study, however, all GGP patients had an additional diagnosis of prior Pso. In a multicenter retrospective investigation, 24/102 (23.5%) GPP patients were additionally diagnosed with PsA (17). In a single-center retrospective observational study from Italy enrolling 140 GPP patients, 8% had additional PsA (18). A large multicenter retrospective cohort study originating from China included 744 GPP patients, of which 3.1% were diagnosed with PsA (19). Hence, previous studies point towards a possible association of GPP with PsA. However, the reported prevalence showed a high variability, ranging from 3.1 to 23.5%. Furthermore, the temporal relationship between this presumed association remains unknown.

Overall, managing PsA poses significant challenges, primarily focusing on disease progression control without joint restoration (20). Therefore, early detection and timely intervention hold considerable potential to preserve patient well-being. Moreover, for Pso patient populations at an elevated risk of developing PsA, treatments with the capacity to prevent its onset may be especially advantageous (2, 21). Thus, we aimed here to determine PsA risk in patients diagnosed with different forms of psoriasis. For this, a retrospective cohort study using a large scale federated database of electronic health records (EHRs) was used.

## 2 Materials and methods

### 2.1 Study design and database

**Figure 1:**
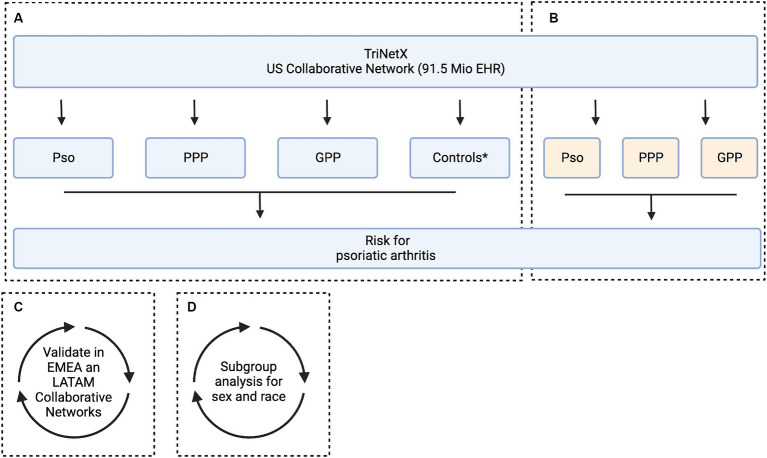
Study flow chart. The aim of this study was to assess the risk of developing psoriatic arthritis among patients diagnosed with psoriasis vulgaris (Pso), pustulosis palmoplantaris (PPP), and generalized pustular psoriasis (GPP). This analysis includes subgroup stratification based on sex and ethnicity. For this, electronic health records (EHRs) indicative of Pso, PPP, and GPP were retrieved from the US Collaborative Network of TriNetX. For each patient cohort, an equally sized, propensity matched control cohort was built. We used two alternative strategies for propensity matching. In Model 1, matching was based on age, sex, and ethnicity, while in Model 2, risk factors for psoriatic arthritis were additionally considered. **(A)** In the initial analysis, the risk to develop psoriatic arthritis was contrasted between each matching case and control group. Subsequently two analyses were performed in parallel. **(B)** To investigate potential variations in the risk of developing psoriatic arthritis across different clinical presentations of psoriasis, we conducted a comparative analysis between Pso, PPP, and GPP. **(C)** To validate our findings, the analyses assessing the risk of developing psoriatic arthritis among patients diagnosed with Pso, PPP, and GPP was repeated in two independent Collaborative Networks within TriNetX, namely EMEA and LATAM.

A global population-based retrospective cohort study with propensity-score matching was performed following previously published protocols (22–26). We retrieved electronic health records (EHRs) from individuals who had been diagnosed with psoriasis vulgaris (Pso), pustulosis palmoplantaris (PPP), or generalized pustular psoriasis (GPP), as well as control subjects who did not have any of these conditions. The grouping criteria were established using ICD-10 codes. Subsequently, we assessed the risk of developing psoriatic arthritis (PsA, ICD-10:L40.5) in the US Collaborative Network, comparing the risk for PsA in individuals with Pso, PPP, or GPP against non-psoriasis controls. To validate our findings, we conducted similar analyses within the EMEA and LATAM Collaborative Networks of TriNetX. Next, we compared the risk for PsA development among individuals with Pso, PPP, and GPP within the US Collaborative Network. Lastly, within the same network, we explored sex- and ethnicity-specific PsA development risks (Figure 1). The data used in this investigation was gathered between February and May 2023 from the US (91 million patients from 57 healthcare organizations), EMEA (15 million patients from 24 healthcare organizations located in the United Kingdom, Sweden, Finland, Spain, Belgium, Germany, Italy, Poland, Hungary, Bulgaria and Israel), and LATAM (26 million patients from 24 healthcare organizations located in Brazil) Collaborative Networks on the TriNetX platform. If indicated, analysis was performed in May 2024. As part of a collaboration between the University Clinic of Schleswig-Holstein (UKSH) and TriNetX, UKSH researchers have access to the TriNetX network.

### 2.2 Ethics statement

This retrospective study is exempt from informed consent. The data reviewed is a secondary analysis of existing data, does not involve intervention or interaction with human subjects, and is de-identified per the de-identification standard defined in Section §164.514(a) of the HIPAA Privacy Rule. The process by which the data is de-identified is attested to through a formal determination by a qualified expert as defined in Section §164.514(b)(1) of the HIPAA Privacy Rule. This formal determination by a qualified expert refreshed on December 2020.

### 2.3 Study population and definition of eligible patients

EHRs of cases and controls were retrieved from the database based on ICD-10 codes. The study included the following groups: individuals with Pso (defined by ICD-10:L40.0, excluding ICD-10:L40.1 and L40.3), PPP (defined by ICD-10:L40.3, excluding ICD-10:L40.0 and L40.1), and GPP (defined by ICD-10:L40.1, excluding ICD10:L40.0 and L40.3), as well as controls who did not exhibit any manifestation of psoriasis in any form (Supplementary Table S1).

### 2.4 Covariates

To allow for better comparability when contrasting the risk for subsequent diagnosis of PsA, propensity-score matching (PSM) was performed by establishing covariate matrixes. We used two different models for propensity matching. In the first model (Model 1), age at index, female sex, and ethnicity were used as variables for propensity matching. In the second model, (Model 2), known risk factors of PsA were additionally used for propensity matching (27, 28). These included: Hyperlipidemia (ICD-10:E78.5), overweight and obesity (ICD-10:E66), disorders of the thyroid gland (ICD-10:E00-07), past (ICD-10:Z87.891) or present (ICD-10:F17) nicotine dependence, depression (ICD-10:F32.A), hyperuricemia (ICD-10:E79.0), and panuveitis (ICD-10:H44.11). Each matrix row order was randomized after data retrieval. A propensity score for each patient was generated by logistic regression using the Python package “scikit-learn.” Matching was performed 1:1 using the greedy nearest neighbor approach with a cut-off distance of 0.1 pooled standard deviations of the logit of the propensity-score. Baseline characteristics were re-evaluated and reported after matching, while differences were compared by *t*-test for continuous variables and *z*-test for binary or categorical variables.

### 2.5 Statistical analysis

For statistical analysis, the index event was set as the diagnosis of any of the indicated psoriatic diseases, or the reported healthcare encounter in the control group. Outcomes any time after the index event were considered in the analysis. Outcomes prior to the diagnosis of each index event were excluded. Relative risks and risk differences were calculated. Survival analyses were performed using the Kaplan–Meier method (KM). KM-curves were compared using the Log-rank test; *p*-values of less than 0.05 were considered significant. Nelson–Aalen plots were utilized to test the proportionality assumption. A univariate Cox proportional hazards regression was used to express hazard ratios (HRs).

## 3 Results

### 3.1 Cohorts

After propensity matching for age at index, gender, and ethnicity (Model 1), we retrieved 35,281, 9,639, and 2,281 EHRs from the US Collaborative Network for Pso, PPP, and GPP, respectively. For each patent cohort, an equally sized, propensity-matched control group was compiled. No significant differences among the matched cases and control groups were noted (Table 1). In the EMEA Collaborative Network, data from 9,834 Pso, 611 PPP, and 250 GPP EHRs were retrieved, with an equal number of propensity-matched controls for each of the patient groups. Again, no differences regarding age, sex, and ethnicity (Model 1) were noted (Supplementary Table S2). In the LATAM Collaborative Network, less EHRs for each patient cohort were present. We retrieved 674, 23, and 16 EHRs for Pso, PPP, and GPP, respectively. Comparison to the equally-sized control groups showed no differences in the parameters used for propensity matching (Supplementary Table S3). To account for potential bias from risk factors of psoriatic arthritis (27, 28), these were included in the propensity matching for Model 2. Because of the relatively low number of retrieved EHRs in the EMEA and LATAM Collaborative Networks of TriNetX, this analysis was solely performed with data retrieved from the US Collaborative Network. After implementing propensity matching in Model 2, we obtained 35,812, 7,788, and 2,408 EHRs from the US Collaborative Network for Pso, PPP, and GPP, respectively. For each patient cohort, an equally-sized, propensity-matched control group was constructed. No discrepancies were observed among cases and control groups for any of the factors used for propensity matching (Table 2).

**Table 1:** Demographics and comorbidities of the study population in the US Collaborative Network (propensity matching Model 1).

Characteristics	Psoriasis vulgaris		Pustulosis palmoplantaris		Generalized pustular psoriasis	
	Cases	Controls	Cases	Controls	Cases	Controls
*N*	35,281	35,281	9,639	9,639	2,281	2,281
Age, years mean ± SD	49.7 ± 19	49.7 ± 19	56.9 ± 20.4	56.9 ± 20.4	36.7 ± 26.8	36.8 ± 26.5
Sex, female (%)	51.8	51.8	77.0	77.0	56.0	56.0
Ethnicity, white (%)	71.4	71.4	74.1	74.1	59.4	59.8

After propensity-matching for the shown variables, no significant differences were observed among all cases and controls. *N*, number; SD, standard deviation.

**Table 2:** Demographics and comorbidities of the study population in the US Collaborative Network (propensity matching Model 2).

Characteristics	Psoriasis vulgaris		Pustulosis palmoplantaris		Generalized pustular psoriasis	
	Cases	Controls	Cases	Controls	Cases	Controls
*N*	35,812	35,812	7,788	7,788	2,408	2,408
Age, years mean ± SD	49.6 ± 19.1	49.6 ± 19.1	55 ± 21.6	55.6 ± 20.5	37.1 ± 26.6	38.6 ± 26.2
Sex, female (%)	51.9	52.0	76.2	75.0	56.4	56.4
Race, white (%)	72.8	72.8	73.4	73.4	60.2	60.6
Hyperlipidemia (%)	23.3	23.3	28.7	30.9	15.2	16.7
Overweight/obesity (%)	19.8	19.8	15.6	16.6	17.0	18.6
Thyroid disorders (%)	15.5	15.6	23.1	23.0	12.0	12.4
Nicotine dependence (%)	12.8	12.9	13.9	13.9	14.4	13.9
History of nicotine dependence (%)	12.3	12.3	12.1	12.9	10.2	10.8
Depression (%)	4.9	5.0	5.8	6.2	3.9	4.0
Hyperuricemia without signs of inflammatory arthritis and tophaceous disease (%)	0.4	0.4	0.36	0.37	0.4	0.4
Panuveitis (%)	0.03	0.03	0.1	0.1	0.4	1.0

After propensity-matching for the shown variables, no significant differences were observed among all cases and controls. *N*, number; SD, standard deviation.

### 3.2 Overall risk for psoriatic arthritis in psoriasis vulgaris, generalized pustular psoriasis and pustulosis palmoplantaris

#### 3.2.1 Contrasting PsA risk between Pso, GPP, and PPP to that of non-psoriasis controls

**Figure 2:**
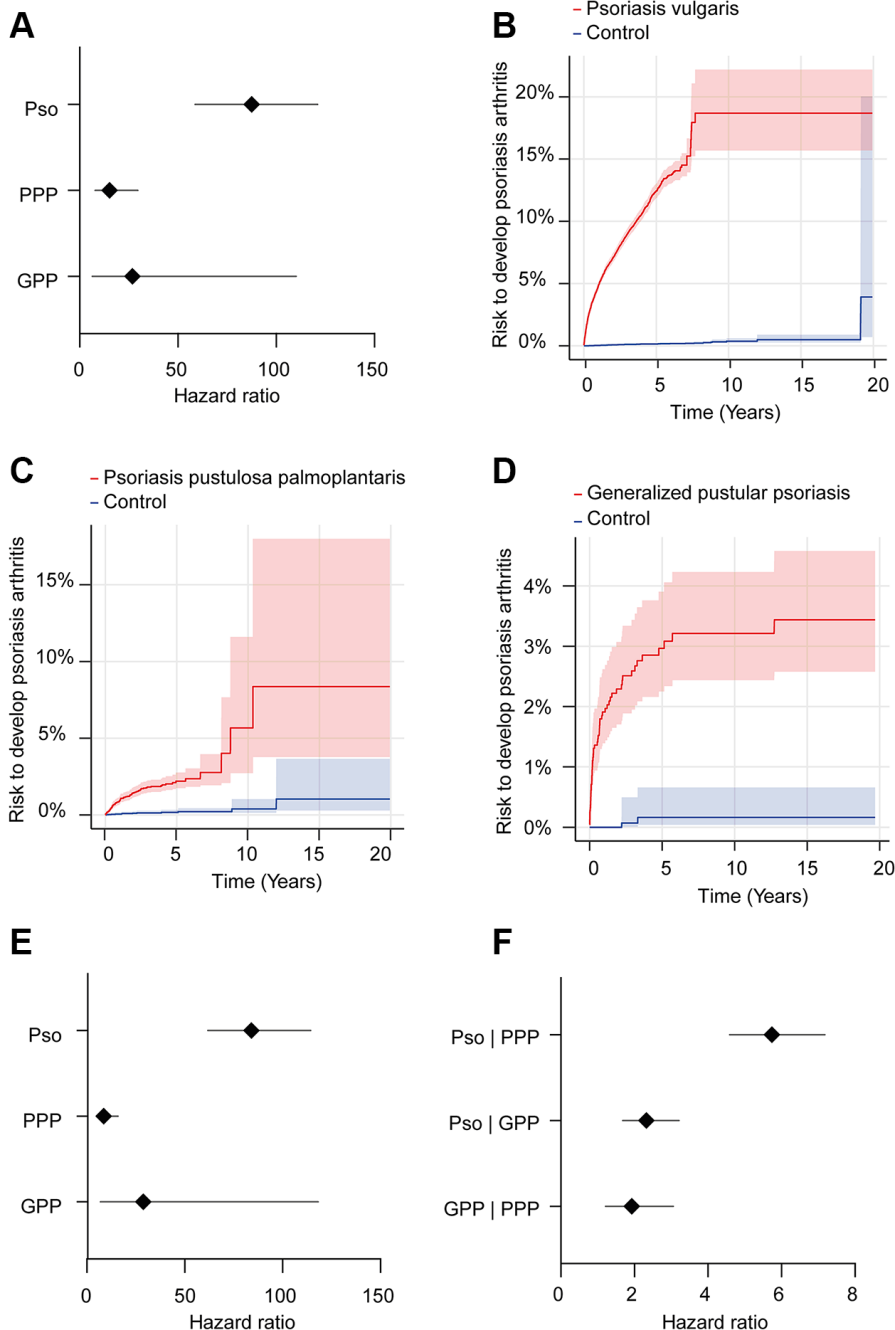
Risk of developing psoriatic arthritis in psoriasis vulgaris, pustulosis palmoplantaris or generalized pustular psoriasis. We assessed the risk of developing psoriatic arthritis within patient cohorts diagnosed with psoriasis vulgaris (Pso), pustulosis palmoplantaris (PPP), or generalized pustular psoriasis (GPP), comparing them to corresponding control groups without any form of psoriasis using electronic health records from the US Collaborative Network of TriNetX. Propensity matching in the initial analysis was confined to age, sex, and ethnicity to allow for better comparability. **(A)** We document an elevated risk of psoriatic arthritis following Pso, PPP, or GPP diagnosis. Diamonds represent hazard ratio (HR) and error bars correspond to the 95% confidence interval (CI). Only significant results are displayed. **(B–D)** Nelson–Aalen plots contrast the risk of psoriatic arthritis of **(B)**. Pso (HR 87.7, CI 63.4–121.1, *p* < 0.0001), **(C)** PPP (HR 15.3, CI 7.9–29.5, *p* < 0.0001), and **(D)** GPP (HR 26.8, CI 6.5–110.1, *p* < 0.0001) to that of the appropriate controls. Solid lines represent the HR and lighter shaded areas represent the CI. **(E)** To address the potential bias introduced by known risk factors for psoriatic arthritis, they were considered for propensity matching. Again, all psoriasis manifestations demonstrated consistent associations with increased psoriatic arthritis risk. Pso maintained the highest risk (HR 84.1, CI 61.8–114.3, *p* < 0.0001), while PPP and GPP displayed substantial risk elevations, with HRs of 8.7 (CI 4.8–15.8, *p* < 0.0001) and 258.8 (CI 7.0–1,118, *p* < 0.0001), respectively. **(F)** To directly compare PsA risks among different psoriasis manifestations, we contrasted this risk between patients with Pso-PPP, Pso-GPP and GPP-PPP. Here, Pso demonstrated a significantly greater risk of developing psoriatic arthritis compared to those with PPP (HR 5.7, CI 4.5–7.1, *p* < 0.0001) and GPP (HR 2.3, CI 1.7–3.2, *p* < 0.0001). In comparison to PPP, we documented a greater risk of psoriatic arthritis development in individuals with GPP (HR 1.9, CI 1.2–3.1, *p* = 0,0042).

We further contrasted the risk to develop psoriatic arthritis in each of the patient cohorts (Pso, PPP, and GPP) to that of the corresponding control groups not affected by any form of psoriasis. For this analysis, we selected the US Collaborative Network of TriNetX because it included considerably more EHRs fulfilling our predefined in- and exclusion criteria. In the initial analysis, propensity matching was limited to age, sex, and ethnicity to maximize the inclusion of a large number of cases. As expected, our analysis revealed an elevated risk of PsA development subsequent to the diagnosis of Pso, PPP, or GPP (Figures 2A–D and Table 3). Notably, significant disparities emerged among the various presentations of psoriasis. The highest risk of PsA was observed in patients with Pso (HR 87.7, 95% CI 63.4–121.1, *p* < 0.0001). While both PPP (HR 15.3, 95% CI 7.9–29.5, *p* < 0.0001) and GPP (HR 26.8, 95% CI 6.5–110.1, *p* < 0.0001) were associated with substantially heightened probabilities of developing PsA compared to their respective control groups, these risks were notably lower than those observed in individuals with Pso.

**Table 3:** Risk of psoriatic arthritis development in varied psoriasis manifestations with propensity matching for age, sex and ethnicity.

Disease	Cases				Controls				Risk difference (95% CI), %	HR (95% CI)	*p*-value
	*N* of eligible participants	*N* of outcomes	Risk, %	Median follow-up (days)	*N* of eligible participants	*N* of outcomes	Risk, %	Median follow-up (days)			
Psoriasis vulgaris	31,018	2,091	6.741	803	35,231	39	0.111	1,209	6.631 (6.349, 6.912)	87.652 (63.435–121.112)	<0.0001
Pustulosis palmoplantaris	9,392	106	1.129	872.5	9,630	11	0.114	1,210	1.014 (0.79, 1.238)	15.303 (7.926–29.543)	<0.0001
Generalized pustular psoriasis	2,175	53	2.437	1,169.5	2,280	10^*^	0.439	1,225	1.998 (1.296, 2.701)	26.824 (6.535–110.115)	<0.0001

CI, confidence interval; HR, hazard ratio; *N*, number; N/A, not applicable. Please note that “Risk %” is used to define “higher” and “lower” risks in subsequent tables comparing the PsA risk of for example, psoriasis vulgaris to that of PPP. Here, psoriasis vulgaris would be indicated as “higher” and PPP as “lower” risk.

Subsequently, to mitigate potential bias introduced by established risk factors for PsA, we conducted a secondary analysis that incorporated these factors into the propensity matching. In this subsequent analysis, we corroborated the findings from the preceding investigation. Specifically, within the model additionally considering known risk factors of PsA, we observed a consistent association between all psoriasis manifestations and an elevated risk of PsA (Figure 2E and Table 4). Notably, Pso exhibited the highest risk (HR 84.1, CI 61.8–114.3, *p* < 0.0001), while PPP and GPP also demonstrated substantially increased risks, with HRs of 8.7 (CI 4.8–15.8, *p* < 0.0001) and 258.8 (CI 7.0–1,118, *p* < 0.0001), respectively.

**Table 4:** Risk of psoriatic arthritis development in varied psoriasis manifestations with propensity matching for age, sex, ethnicity, and known risk factors of psoriatic arthritis.

Disease	Cases				Controls				Risk difference (95% CI), %	HR (95% CI)	*p*-value
	*N* of eligible participants	*N* of outcomes	Risk, %	Median follow-up (days)	*N* of eligible participants	*N* of outcomes	Risk, %	Median follow-up (days)			
Psoriasis vulgaris	31,237	2,198	7.037	803	35,744	44	0.123	1,227	6.913 (6.627, 7.199)	84.133 (61.883–114.385)	<0.0001
Pustulosis palmoplantaris	7,565	102	1.348	872.5	7,770	12	0.154	1,267	1.194 (0.92, 1.468)	8.73 (4.804–15.865)	<0.0001
Generalized pustular psoriasis	2,291	55	2.401	1,169.5	2,406	10^*^	0.416	1,303.5	1.985 (1.308, 2.663)	28.819 (7.026–118.202)	<0.0001

CI, confidence interval; HR, hazard ratio; *N*, number; N/A, not applicable.

To independently validate our findings, we conducted an investigation into the risk of PsA in cohorts of Pso, PPP, and GPP obtained from the EMEA and LATAM Collaborative Networks. Due to the limited number of EHRs available in these Networks, our analysis was confined to Model 1. Within the EMEA Collaborative Network, Pso, consistent with previous findings, was associated with an increase in the risk of subsequent PsA (HR 181.8, CI 58.4–565.3, *p* < 0.0001). For PPP, HR and CI could not be calculated due to the absence of outcomes in the control group, nevertheless we observed a statistically significant increased risk for PsA (*p* < 0.0001). Conversely, GPP did not demonstrate a statistically significant risk of PsA (Supplementary Table S4). In the LATAM Collaborative Network, Pso also displayed an increased risk of PsA, with an HR of 20.8 (CI 2.8–154.5, *p* < 0.0001). However, data regarding PPP and GPP cannot be considered due to the insufficient number of EHRs in both cases and controls, as well as the limited number of outcomes (Supplementary Table S5).

#### 3.2.2 Contrasting PsA risk between Pso, PPP and GPP

Our earlier findings highlighted a notable disparity in the risk of subsequent PsA development, with Pso exhibiting the highest risk while PPP displayed the lowest risk. To perform a direct comparison of PsA risk among different psoriasis manifestations, we conducted pairwise comparisons between Pso and PPP, Pso and GPP, and GPP and PPP. These comparisons were performed after propensity matching the respective cohorts for age, sex, and ethnicity (Table 5). In accordance with our descriptive observations, individuals diagnosed with Pso demonstrated a significantly greater risk of developing PsA compared to those with PPP (HR 5.7, CI 4.5–7.1, *p* < 0.0001) and GPP (HR 2.3, CI 1.7–3.2, *p* < 0.0001, Figure 2F and Table 6). The direct comparison of psoriatic arthritis risk between GPP and PPP also mirrored our descriptive findings. In comparison to PPP, we documented a greater risk of PsA development in individuals with GPP (HR 1.9, CI 1.2–3.1, *p* = 0,0042, Figure 2F and Table 6).

**Table 5:** Demographics of the study population in relative association analysis in the US Collaborative Network.

Diseases compared	Psoriasis vulgaris	Pustulosis palmoplantaris	Psoriasis vulgaris	Generalized pustular psoriasis	Generalized pustular psoriasis	Pustulosis palmoplantaris
*Descriptive risk difference*	Higher^*^	Lower^*^	Higher^*^	Lower^*^	Higher^*^	Lower^*^
*N*	7,673	7,673	2,405	2,405	2,281	2,281
Age, years mean ± SD	56.8 ± 19.9	55.3 ± 21.4	39.4 ± 25.2	38.7 ± 26.0	40.2 ± 25.8	41.2 ± 24.9
Sex, female (%)	74.6	76.3	58.7	55.6	59.8	54.8
Ethnicity, white (%)	75.5	73.4	59.0	62.1	60.8	60.8

After propensity-matching for the shown variables, no significant differences were observed among all groups with the higher and lower relative association strengths. *N*, number; SD, standard deviation. ^*^The terms “higher” and “lower” pertain to the descriptive comparison of the risk of developing psoriatic arthritis, as indicated in Table 3.

**Table 6:** Comparative risk of psoriatic arthritis development amongst varied psoriasis manifestations.

Diseases		Higher descriptive risk				Lower descriptive risk				Risk difference (95% CI), %	HR (95% CI)	*p*-value
Higher descriptive risk	Lower descriptive risk	*N* of eligible participants	*N* of outcomes	Risk, %	Median follow-up (days)	*N* of eligible participants	*N* of outcomes	Risk, %	Median follow-up (days)			
Psoriasis vulgaris	Pustulosis palmoplantaris	6,677	462	6.919	826	7,452	93	1.248	872.5	5.671 (5.012, 6.33)	5.744 (4.593–7.182)	<0.0001
Psoriasis vulgaris	Generalized pustular psoriasis	2,180	105	4.817	804	2,283	61	2.672	1,144	2.145 (1.029, 3.261)	2.332 (1.691–3.216)	<0.0001
Generalized pustular psoriasis	Pustulosis palmoplantaris	2,159	60	2.779	1,127	2,221	27	1.216	833	1.563 (0.734, 2.393)	1.933 (1.221–3.059)	0.0042

*N*, number; *SD*, standard deviation.

### 3.3 Sex-specific risks for psoriatic arthritis in psoriasis vulgaris, generalized pustular psoriasis and pustulosis palmoplantaris

#### 3.3.1 Contrasting PsA risk between Pso, PPP, and GPP to that of non-psoriasis controls

To explore the possibility of sex-specific differences for PsA risk subsequent to diagnosis of Pso, PPP, or GPP, we conducted a comparative analysis of this risk within subgroups defined by female or male sex among cases and controls. Due to the limited availability of EHRs in the EMEA and LATAM Collaborative Networks, these analyses were conducted using the US Collaborative Network. As our previous analyses yielded consistent results between both models for propensity matching, we adopted Model 1 (excluding sex) for propensity matching.

**Figure 3:**
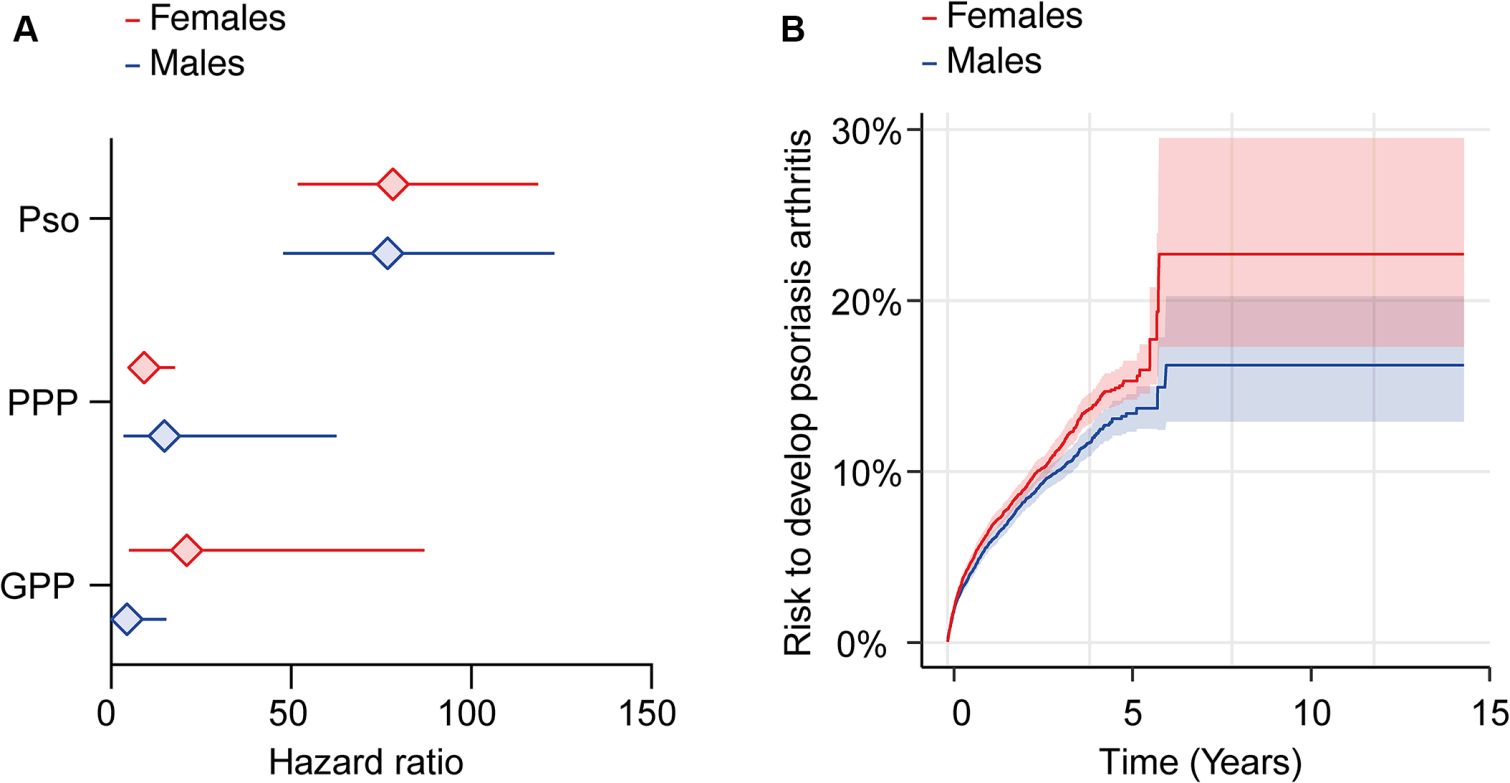
Sex-specific risks for the development of psoriatic arthritis following a diagnosis of psoriasis vulgaris, pustulosis palmoplantaris or generalized pustular psoriasis. We assessed the risk of developing psoriatic arthritis within sex-stratified (female or male) patient cohorts diagnosed with psoriasis vulgaris (Pso), pustulosis palmoplantaris (PPP), or generalized pustular psoriasis (GPP), comparing them to corresponding control groups without any form of psoriasis using electronic health records from the US Collaborative Network of TriNetX. Propensity matching in the initial analysis was confined to age and ethnicity to allow for better comparability. **(A)** We document an elevated risk of psoriatic arthritis following Pso, PPP, or GPP diagnosis in both female (red) and male (blue) patients. Diamonds represent hazard ratio (HR) and error bars correspond to the 95% confidence interval (CI). Only significant results are displayed. **(B)** The Nelson–Aalen plot contrasts the risk of psoriatic arthritis in female (red) and male (blue) Pso patients. Compared to male Pso patients, the risk of psoriatic arthritis in their female counterparts is significantly greater (HR 1.1, CI 1.1–1.2, *p* = 0.002). Solid lines represent the HR and lighter shaded areas represent the CI.

Depending on psoriasis manifestation and sex, we retrieved 1,038 to 16,787 cases and controls per group. Following propensity matching, cases and controls for all investigations were similar in regard to age and White ethnic proportion (Supplementary Table S6). The analysis stratified to EHRs indicating female sex demonstrated an increased risk of PsA after the diagnosis of Pso, PPP, or GPP (Figure 3A and Supplementary Table S7). As observed in the non-stratified analysis, striking inequalities were present among the various presentations of psoriasis. More specifically, the highest risk of PsA was observed in female patients with Pso (HR 78.4, CI 51.8–118.6, *p* < 0.0001). In female PPP (HR 9.3, CI 4.8–17.8, *p* < 0.0001) and GPP (HR 21.0, CI 5.1–87.1, *p* < 0.0001) patients the risk of PsA was also increased, but to a lesser extent compared to Pso (Figure 3A and Supplementary Table S7). Risk for PsA was also increased in male individuals diagnosed with Pso (HR 76.7, CI 47.8–123.1, *p* < 0.0001), PPP (HR 14.8, CI 3.5–62.5, *p* < 0.0001), and GPP (HR 4.4, CI 1.3–15.3, *p* < 0.0001, Figure 3A and Supplementary Table S8). Considering the discernible variations in psoriatic arthritis risk among female and male patients diagnosed with Pso, PPP, or GPP, we proceeded to contrast the relative risk of PsA between female and male Pso patients. The demographics after propensity matching for age and ethnicity for this comparison are depicted in Supplementary Table S9. The cumulative risk of developing PsA was 7.8% among female patients diagnosed with Pso, in contrast to 6.6% among their male counterparts. Hence, female Pso patients exhibit a marginally higher, yet statistically significant increased risk to develop psoriatic arthritis (HR 1.1, CI 1.1–1.2, *p* = 0.002, Figure 3B and Supplementary Table S10). Because of the limited number of cases and outcomes, we refrained from conducting a similar analysis for PPP and GPP.

#### 3.3.2 Contrasting PsA risk between psoriasis vulgaris, GPP and PPP

Likewise, in respect to the comparisons made for the different clinical manifestations of psoriasis, we also compared the PsA risk between Pso, GPP and PPP here. Our sex-stratified analyses corroborated the previous non-sex-stratified results. Specifically, in EHRs stratified for female sex, the PsA risk was highest in Pso, followed by GPP, and lowest in PPP. These differences were statistically significant when directly comparing the PsA risk between any two clinical manifestations of psoriasis. Similar findings were observed in EHRs stratified by male sex. Detailed demographics and results can be found in Supplementary Tables S11–S14.

### 3.4 Ethnicity-specific risk for psoriatic arthritis in psoriasis vulgaris, generalized pustular psoriasis and pustulosis palmoplantaris

#### 3.4.1 Contrasting PsA risk between Pso, GPP, and PPP to that of non-psoriasis controls

Next, to investigate potential ethnic disparities in the likelihood of developing PsA following diagnoses of Pso, PPP, or GPP, we carried out a comparative risk analysis within subgroups categorized by either White or Black or African American ethnicity among both cases and controls. Due to the restricted number of EHRs fulfilling our in- and exclusion criteria in the EMEA and LATAM Collaborative Networks, along with limited ethnicity coding in these networks, we limited these analyses to the US Collaborative network. Given the consistent outcomes observed in our earlier analyses using both models for propensity matching, we used Model 1 (excluding ethnicity) for propensity matching.

**Figure 4:**
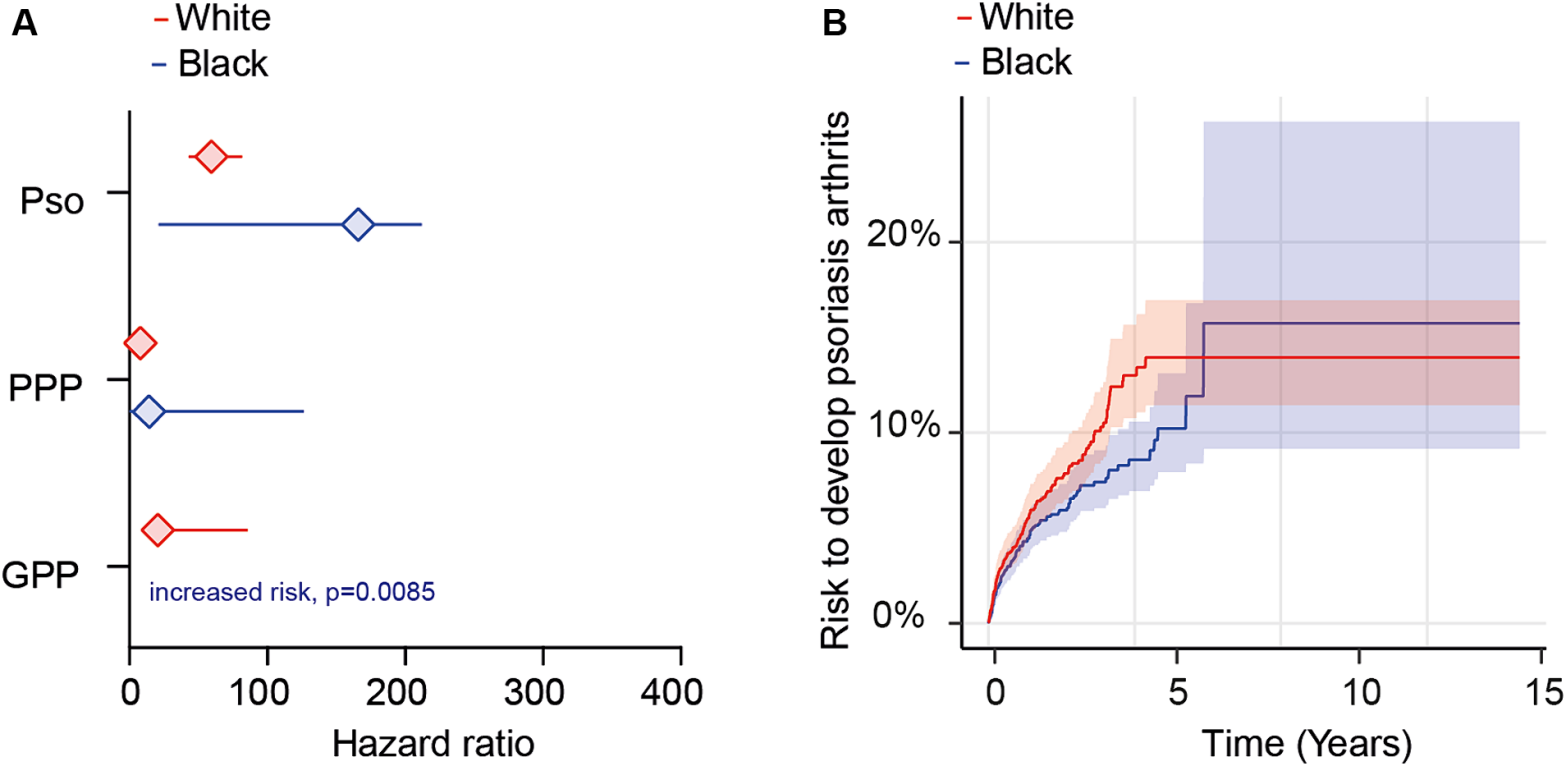
Ethnicity-specific risks for the development of psoriatic arthritis following a diagnosis of psoriasis vulgaris, pustulosis palmoplantaris or generalized pustular psoriasis. We assessed the risk of developing psoriatic arthritis within ethnicity-stratified (Black or African American/White) patient cohorts diagnosed with psoriasis vulgaris (Pso), pustulosis palmoplantaris (PPP), or generalized pustular psoriasis (GPP), comparing them to corresponding control groups without any form of psoriasis using electronic health records from the US Collaborative Network of TriNetX. Propensity matching in the initial analysis was confined to age and sex to allow for better comparability. **(A)** We document an elevated risk of psoriatic arthritis following Pso, PPP, or GPP diagnosis in both Black or African American (blue) and White (red) patients. Diamonds represent hazard ratio (HR) and error bars correspond to the 95% confidence interval (CI). Only significant results are displayed. **(B)** The Nelson–Aalen plot contrasts the risk of psoriatic arthritis in Black or African American (red) and White (blue) Pso patients. Compared to Black or African American Pso patients, the risk of psoriatic arthritis in their White counterparts is significantly greater (HR 1.3, CI 1.1–3.2, *p* = 0.024). Solid lines represent the HR and lighter shaded areas represent the CI.

Depending on psoriasis manifestation and ethnicity, we retrieved between 486 and 23,043 cases and controls per group. Following propensity matching, cases and controls for all investigations were similar regarding age and sex (Supplementary Table S15). When we stratified the analysis based on EHRs indicating Black or African American ethnicity, we observed an elevated risk of PsA following the diagnosis of Pso, PPP, or GPP (Figure 4A and Supplementary Table S16). Similar to our findings in the non-stratified analysis, disparities were evident among the different psoriasis presentations. Specifically, the highest risk of PsA was noted in Black or African American patients with Pso (HR 167.8, CI 23.2–1,214.2, *p* < 0.0001). Black or African American PPP patients also exhibited an increased risk, albeit to a lesser extent compared to Pso (HR 16.1, CI 2.0–128.8, *p* = 0.0005). For GPP, HR and CI could not be calculated due to the absence of outcomes in the control group; nevertheless, a statistically significant increased risk for PsA was observed (*p* < 0.0085, Figure 4A and Supplementary Table S16). Similarly, the risk for PsA was elevated among White individuals diagnosed with Pso (HR 59.4, CI 43.1–81.7, *p* < 0.0001), PPP (HR 8.0, CI 4.3–14.8, *p* < 0.0001), and GPP (HR 20.7, CI 5.0–85.6, *p* < 0.0001, Figure 4A and Supplementary Table S17). Given the noticeable differences in PsA risk among Black or African American and White patients diagnosed with Pso, PPP, or GPP, we proceeded to compare its relative risk among these two ethnic groups. The demographic characteristics after propensity matching for age and sex for this comparison are provided in Supplementary Table S18. The cumulative risk of developing PsA was 5.5% among Black or African American patients diagnosed with Pso, in contrast to 7.2% among their White propensity matched controls. Thus, White Pso patients exhibited a slightly higher, yet statistically significant, increased risk of developing psoriatic arthritis (HR 1.3, CI 1.04–1.7, *p* = 0.024, Figure 4B and Supplementary Table S19). Due to the limited number of cases and outcomes, we refrained from conducting a similar analysis for PPP and GPP.

#### 3.4.2 Contrasting PsA risk between psoriasis vulgaris, GPP and PPP

The ethnicity-stratified analyses regarding PsA risk in EHRs indicating Black or African American, or alternatively White ethnicity, corroborated the previous non-ethnicity-stratified results. Specifically, in EHRs stratified for Black or African American ethnicity, the PsA risk was highest in Pso, followed by GPP, and lowest in PPP. These differences were statistically significant when directly comparing the PsA risk between any two clinical manifestations of psoriasis. Similar findings were observed in EHRs stratified by White ethnicity. However, due to the low sample size (*N* = 428) and low number of outcomes (*n* = 10), no significant difference was found between Black and White EHRs when contrasting GPP and PPP. Detailed demographics and results can be found in Supplementary Tables S20–S23.

## 4 Discussion

We demonstrate here that psoriasis patients’ risk to develop PsA varies greatly depending on their disease variant, sex, and ethnicity. Specifically, the likelihood of PsA development ranged from 1.1 to 7.4%. The lowest risk for PsA was observed in Black or African American PPP patients, while the highest risk was seen in female Pso patients. Similarly, striking differences were noted when focusing on sex- and ethnicity-stratified cohorts within Pso, PPP, and GPP patients. For example, the likelihood of PsA development ranged from 5.5% (Black or African American) to 7.4% (female) in Pso patients. PsA prevalence following PPP diagnosis ranged from 1.1% (Black or African American) to 1.5% (male). In GPP, the prevalence of subsequent psoriatic arthritis ranged from 1.5% (male) to 3.2% (female). These results underscore the importance of stringently implementing sex- and ethnic-specific analyses in epidemiology research. In the past, ethnicity and sex have largely been understudied epidemiologically, especially for the topic addressed herein (11, 12, 29).

### 4.1 Impact of sex on PsA risk

Overall, the previously reported PsA risk in individual studies shows high variability in the male-to-female ratio, spanning from 0.42 to 2.75 (12). In a prospective, population-based annual incidence study of early arthritis, including PsA, from Sweden, 8 out of 100,000 women and 3 out of 100,000 men were diagnosed with PsA. This translates into the highest reported risk for female psoriasis patients to develop PsA. The relatively low number of PsA patients, however, may have introduced a relevant bias (30). In addition to this study, two additional investigations supported the notion of a higher PsA risk in females compared to males (12). However, these results were contradicted by an Argentinian study which evaluated the risk of PsA development in around 140,000 people presenting to a university hospital-based health management organization in Buenos Aires over a 6-year period. Here, a total of 35 patients developed PsA, including 12 female and 23 male patients (31). The greater male PsA risk observed here has also been reported in several other studies. Here again, small sample size in all those studies, ranging from 147 to 329 PsA patients, should be considered as a potential source of bias (32–35). Lastly, a study from the US showed no sex differences in PsA prevalence in a cohort of 65 patients diagnosed with this disease (36). Our study retrieved 31,018 EHRs with a diagnosis of Pso, of which 2,091 were subsequently diagnosed with PsA in the cohort not stratified for sex or ethnicity (Table 3). In our sex-stratified analysis, 1,082 of the 14,453 female Pso patients were diagnosed with PsA, while only 894 of the 13,552 male Pso patients received a PsA diagnosis (Supplementary Tables S9, S10). Consequently, any potential bias resulting from the limited number of outcomes in our study would be relatively low. Additionally, upon direct comparison of PsA risk between male and female Pso patients, a higher risk was evident in the latter group (Supplementary Table S12). Thus, overall, females Pso patients exhibit a heightened risk to develop PsA. This trend remains consistent for GPP, with 3.2% of female GPP patients subsequently developing PsA. However, this pattern is not seen in PPP, where the risk of PsA remained relatively similar between female and male PPP patients. Nevertheless, it is imperative to exercise caution in interpreting these findings due to the relatively low number of PsA cases within the PPP and GPP groups (Supplementary Tables S9, S10).

In regard to clinical translation, our study data enables the stratification of psoriasis patients into distinct PsA risk groups. Within high-risk categories, leveraging established PsA risk factors alongside the potential integration of novel biomarkers holds promise for enhancing PsA risk prediction. The findings support the implementation of a more nuanced approach involving frequent PsA evaluations and earlier initiation of targeted biological treatments, which may potentially reduce the likelihood of PsA development (2, 21).

### 4.2 Limitations

This study has several limitations to be appreciated. Primarily, cohorts were identified using ICD-10 codes, which to a certain degree include coding inaccuracies. This issue likely applies to our study because the observed prevalence of PsA was lower than has been reported elsewhere. Thus, PsA is most likely under-diagnosed within the HCOs contributing data to TriNetX (9). As this is a systemic issue, the relative differences reported here remain valid. In defining psoriasis cohorts by including one variant of the disease while simultaneously excluding any other variants, we partially addressed this bias. We were also unable to consider the severity of psoriasis, since ICD-10:L40.70, the code for “Moderate to severe psoriasis,” was not accessible. Furthermore, this study did not include medications because we aimed to include as many EHRs as possible. Thus, the potential impact of medications has not been addressed and will be the focus of subsequent endeavors. When comparing PsA risk of psoriasis patients versus non-psoriasis controls, an assessment bias (i.e., patients with psoriasis are more often screened for the presence of PsA) could potentially confound the results. Lastly, due to the study’s retrospective design, differences in PsA risks found here cannot be causally attributed to underlying psoriasis.

## 5 Conclusion

In conclusion, our data provides comprehensive insights into the sex- and ethnicity-specific risks associated with development of PsA after a diagnosis of Pso, PPP, or GPP. Our findings underscore the opportunity for more precise PsA monitoring tailored to individual patient characteristics and specific psoriasis variants.

## Data availability statement

The original contributions presented in the study are included in the article/Supplementary material, further inquiries can be directed to the corresponding author.

## Ethics statement

Ethical approval was not required for the study involving humans in accordance with the local legislation and institutional requirements. Written informed consent to participate in this study was not required from the participants or the participants’ legal guardians/next of kin in accordance with the national legislation and the institutional requirements.

## Author contributions

BG: Investigation, Writing – original draft, Writing – review & editing. KB: Visualization, Writing – review & editing. AV: Visualization, Writing – review & editing. ML: Investigation, Writing – review & editing. HZ: Investigation, Writing – review & editing. DD: Investigation, Writing – review & editing. DT: Investigation, Supervision, Writing – review & editing. KK: Conceptualization, Investigation, Writing – review & editing. RL: Conceptualization, Data curation, Funding acquisition, Investigation, Methodology, Project administration, Supervision, Writing – original draft.
