# Supplementary material for: Differential risks of psoriatic arthritis development in patients with varied psoriasis manifestations: a sex- and ethnicity-specific analysis
Source: Front Med (Lausanne). 2024 Jun 21;11:1385491. doi: 10.3389/fmed.2024.1385491 (PMC11224429; doi:10.3389/fmed.2024.1385491)
Supplement: Supplementary file 1 [file Table_1.docx]

**Supplemental Tables**

| **Psoriasis Vulgaris** | | **Pustulosis Palmoplantaris** | | **Generalized Pustular Psoriasis** | | **Controls** |
| --- | --- | --- | --- | --- | --- | --- |
| **Must have** | **Cannot have** | **Must have** | **Cannot have** | **Must have** | **Cannot have** | **Must only have** |
| L40.0 | L40.1  L40.3 | L40.3 | L40.0  L40.1 | L40.1 | L40.0  L40.3 | Z00* |

**Supplemental Table 1.** Diagnostic ICD10 codes for definition of cohorts.

| **Characteristics** | **Psoriasis Vulgaris** | | **Pustulosis Palmoplantaris** | | **Generalized Pustular Psoriasis** | |
| --- | --- | --- | --- | --- | --- | --- |
|  | **Cases** | **Controls** | **Cases** | **Controls** | **Cases** | **Controls** |
| ***N*** | 9,834 | 9,834 | 611 | 611 | 250 | 250 |
| **Age, years**  **Mean ± SD** | 47.7 ± 18.4 | 47.7 ± 18.4 | 53.6 ± 16.4 | 53.6 ± 16.4 | 56.3 ± 21.1 | 56.3 ± 21.1 |
| **Sex, female (%)** | 45.7 | 45.7 | 73.9 | 74.4 | 61.6 | 62.4 |
| **Ethnicity*, white (%)** | 0.4 | 0.4 | 5.0 | 5.0 | 9.2 | 9.2 |

**Supplemental Table 2.** Demographics and comorbidities of the study population in the EMEA Collaborative Network. After propensity-matching for the shown variables, no significant differences were observed among all cases and controls. Abbreviations: *N*, number; *SD*, standard deviation. *Ethnicity lacks comprehensive documentation within the EMEA Collaborative Network. Hence the proportion of EHRs indicating “White” is low.

| **Characteristics** | **Psoriasis Vulgaris** | | **Pustulosis Palmoplantaris** | | **Generalized Pustular Psoriasis** | |
| --- | --- | --- | --- | --- | --- | --- |
|  | **Cases*** | **Controls*** | **Cases*** | **Controls*** | **Cases*** | **Controls*** |
| ***N*** | 674 | 674 | 23 | 23 | 16 | 16 |
| **Age, years**  **Mean ± SD** | 44.9 ± 18.2 | 44.9 ± 18.2 | 35.7 ± 27.7 | 35.7 ± 27.7 | 40.9 ± 18.4 | 40.9 ± 18.4 |
| **Sex, female (%)** | 52.7 | 52.7 | 65.2 | 65.2 | 62.5 | 62.5 |
| **Ethnicity*, white (%)** | 12.5 | 12.5 | 0 | 0 | 62.5 | 62.5 |

**Supplemental Table 3.** Demographics and comorbidities of the study population in the LATAM Collaborative Network. After propensity-matching for the shown variables, no significant differences were observed among all cases and controls. Abbreviations: *N*, number; *SD*, standard deviation. *Ethnicity lacks comprehensive documentation within the EMEA Collaborative Network. Hence the proportion of EHRs indicating “White” is low.

|  | **Cases** | | | **Controls** | | |  |  |  |
| --- | --- | --- | --- | --- | --- | --- | --- | --- | --- |
| **Disease** | ***N* of eligible participants** | ***N* of outcomes** | **Risk, %** | ***N* of eligible participants** | ***N* of outcomes** | **Risk, %** | **Risk difference (95% CI), %** | **HR**  **(95% CI)** | ***P* value** |
| Psoriasis Vulgaris | 9,050 | 626 | 6.917 | 9,829 | 10 | 0.102 | 6.815  (6.289, 7.342) | 181.829  (58.484-565.316) | < 0.0001 |
| Pustulosis Palmoplantaris | 583 | 20 | 3.431 | 611 | 0 | 0 | 3.431  (1.953,4.908) | N/A | < 0.0001 |
| Generalized Pustular Psoriasis | 228 | 10* | 4.386 | 250 | 0 | 0 | 4.386  (1.728,7.044) | N/A | 0.1407 |

**Supplemental Table 4.** Risk of psoriatic arthritis development in varied psoriasis manifestations in the EMEA Collaborative Network. Abbreviations: *CI*, confidence interval; *HR,* hazard ratio; *N*, number; *N/A*, not applicable. *1-10 outcomes are by default displayed by 10 by TriNetX.

|  | **Cases** | | | **Controls** | | |  |  |  |
| --- | --- | --- | --- | --- | --- | --- | --- | --- | --- |
| **Disease** | **N of eligible participants** | **N of outcomes** | **Risk, %** | **N of eligible participants** | **N of outcomes** | **Risk, %** | **Risk difference (95% CI), %** | **HR**  **(95% CI)** | **P value** |
| Psoriasis Vulgaris | 645 | 23 | 3.566 | 674 | 10 | 1.484 | 2.082%  (0.385,3.78) | 20.863  (2.816-154.556 | < 0.0001 |
| Pustulosis Palmoplantaris | 22 | 10* | 45.455 | 23 | 0 | 0 | 45.455  (24.647,66.262) | N/A | 0.3980 |
| Generalized Pustular Psoriasis | 15 | 10* | 66.667 | 16 | 0 | 0 | 66.667%  (42.81,90.523) | N/A | 0.0591 |

**Supplemental Table 5.** Risk of psoriatic arthritis development in varied psoriasis manifestations in the LATAM Collaborative Network. For PPP and GPP, please note that both the number of EHRs and the number of outcomes for cases and controls is too low to allow valid conclusions. They are thus displayed in gray. Abbreviations: *CI*, confidence interval; *HR,* hazard ratio; *N*, number; *N/A*, not applicable. *1-10 outcomes are by default displayed by 10 by TriNetX.

| **Characteristics** | **Psoriasis Vulgaris** | | | | **Pustulosis Palmoplantaris** | | | | **Generalized Pustular Psoriasis** | | | |
| --- | --- | --- | --- | --- | --- | --- | --- | --- | --- | --- | --- | --- |
|  | **Female** | | **Male** | | **Female** | | **Male** | | **Female** | | **Male** | |
|  | ***Cases*** | ***Controls*** | ***Cases*** | ***Controls*** | ***Cases*** | ***Controls*** | ***Cases*** | ***Controls*** | ***Cases*** | ***Controls*** | ***Cases*** | ***Controls*** |
| ***N*** | 16,787 | 16,787 | 15,326 | 15,326 | 5,852 | 5,852 | 1,683 | 1,683 | 1,342 | 1,342 | 1,038 | 1,038 |
| **Age, years**  **Mean ± SD** | 49.9 +/-  19.6 | 49.9 +/-  19.6 | 49.8 +/-  18.8 | 49.8 +/-  18.8 | 58.2 +/-  18.9 | 58.2 +/-  18.9 | 45.4 +/-  26.2 | 45.4 +/-  26.2 | 39.2 +/- 25.8 | 39.3 +/-  25.6 | 34.2 +/- 27.3 | 34.2 +/-  27.3 |
| **Sex, female (%)** | 100 | 100 | 0 | 0 | 100 | 100 | 0 | 0 | 100 | 100 | 0 | 0 |
| **Ethnicity, white (%)** | 72.3 | 72.3 | 71.1 | 71.1 | 75.8 | 75.8 | 64.9 | 64.9 | 59.9 | 60.7 | 60.4 | 60.2 |

**Supplemental Table 6.** Demographics of the study population in subgroup analysis for sex. After propensity-matching for the shown variables, no significant differences were observed among all cases and controls. Abbreviations: *N*, number; *SD*, standard deviation.

|  | **Cases** | | | **Controls** | | |  |  |  |
| --- | --- | --- | --- | --- | --- | --- | --- | --- | --- |
| **Disease** | ***N* of eligible participants** | ***N* of outcomes** | **Risk, %** | ***N* of eligible participants** | ***N* of outcomes** | **Risk, %** | **Risk difference (95% CI), %** | **HR**  **(95% CI)** | ***P* value** |
| Psoriasis Vulgaris | 14,453 | 1,082 | 7.486 | 16,776 | 25 | 0.149 | 7.337  (6.904, 7.77) | 78.37  (51.779-118.617) | < 0.0001 |
| Pustulosis Palmoplantaris | 5,677 | 68 | 1.198 | 5,839 | 12 | 0.206 | 0.992  (0.686, 1.298) | 9.287  (4.835-17.838) | < 0.0001 |
| Generalized Pustular Psoriasis | 1,261 | 40 | 3.172 | 1,341 | 10* | 0.746 | 2.426  (1.355, 3.498) | 21.041  (5.085-87.064) | < 0.0001 |

**Supplemental Table 7.** Risk of psoriatic arthritis development in varied psoriasis manifestations in female cases and controls. Abbreviations: *CI*, confidence interval; *HR,* hazard ratio; *N*, number. *1-10 outcomes are by default displayed by 10 by TriNetX.

|  | **Cases** | | | **Controls** | | |  |  |  |
| --- | --- | --- | --- | --- | --- | --- | --- | --- | --- |
| **Disease** | ***N* of eligible participants** | ***N* of outcomes** | **Risk, %** | ***N* of eligible participants** | ***N* of outcomes** | **Risk, %** | **Risk difference (95% CI), %** | **HR**  **(95% CI)** | ***P* value** |
| Psoriasis Vulgaris | 13,552 | 894 | 6.597 | 15,294 | 18 | 0.118 | 6.479  (6.058, 6.901) | 76.732  (47.816-123.133) | < 0.0001 |
| Pustulosis Palmoplantaris | 1,638 | 25 | 1.526 | 1,682 | 10* | 0.595 | 0.932  (0.234, 1.63) | 14.798  (3.503-62.52) | < 0.0001 |
| Generalized Pustular Psoriasis | 1,007 | 15 | 1.49 | 1,036 | 10* | 0.965 | 0.524  (-0.432, 1.48) | 4.414  (1.269-15.348) | 0.0107 |

**Supplemental Table 8.** Risk of psoriatic arthritis development in varied psoriasis manifestations in male cases and controls. Abbreviations: *CI*, confidence interval; *HR,* hazard ratio; *N*, number. *1-10 outcomes are by default displayed by 10 by TriNetX.

| **Characteristics** | **Psoriasis vulgaris** | |
| --- | --- | --- |
|  | **Women** | **Men** |
| ***Descriptive risk difference*** | ***Higher**** | ***Lower**** |
| ***N*** | 17,092 | 17,092 |
| **Age, years**  **Mean ± SD** | 49.6 ± 18.7 | 49.6 ± 18.7 |
| **Sex, female (%)** | 100 | 0 |
| **Ethnicity, white (%)** | 72.9 | 72.9 |

**Supplemental Table 9.** Demographics of the study population in subgroup relative association analysis (sex). After propensity-matching for the shown variables, no significant differences were observed among all groups with the higher and lower relative association strengths, except for sex. Abbreviations: *N*, number; *SD*, standard deviation. *The terms "Higher" and "Lower" pertain to the descriptive comparison of the risk of developing psoriatic arthritis as indicated in **Supplemental** **Tables 7-8**.

| **Subgroups** | | **Higher descriptive risk** | | | **Lower descriptive risk** | | |  |  |  |
| --- | --- | --- | --- | --- | --- | --- | --- | --- | --- | --- |
| **Higher descriptive risk** | **Lower descriptive risk** | ***N* of eligible participants** | ***N* of outcomes** | **Risk, %** | ***N* of eligible participants** | ***N* of outcomes** | **Risk, %** | **Risk difference (95% CI), %** | **HR**  **(95% CI)** | ***P* value** |
| Women | Men | 14,652 | 1,143 | 7.801 | 15,107 | 998 | 6.606 | 1.195  (0.607, 1.783) | 1.143  (1.05-1.244) | 0.0020 |

**Supplemental Table 10.** Risk of psoriatic arthritis development in female compared to male psoriasis vulgaris patients. Abbreviations: *CI*, confidence interval; *HR,* hazard ratio; *N*, number; *N/A*, not applicable.

| **Diseases Compared** | Psoriasis Vulgaris | Pustulosis Palmoplantaris | Psoriasis Vulgaris | Generalized Pustular Psoriasis | Generalized Pustular Psoriasis | Pustulosis Palmoplantaris |
| --- | --- | --- | --- | --- | --- | --- |
| ***Descriptive risk difference*** | ***Higher**** | ***Lower**** | ***Higher**** | ***Lower**** | ***Higher**** | ***Lower**** |
| ***N*** | 9,016 | 9,016 | 1,571 | 1,571 | 1,528 | 1,528 |
| **Age, years**  **Mean ± SD** | 58.8 ± 17.8 | 58.8 ± 18.1 | 43.8 ± 23.1 | 43.1 ± 23.8 | 44 ± 23.6 | 44.1 ± 23.5 |
| **Sex, female (%)** | 100 | 100 | 100 | 100 | 100 | 100 |
| **Ethnicity, white (%)** | 78.638^§^ | 77.252^§^ | 63.717 | 65.627 | 65.838 | 64.987 |

**Table 11.** Demographics of the study population in relative association analysis in the US Collaborative Network for EHRs indicating female sex. After propensity-matching for the shown variables, no significant differences were observed among all groups with the higher and lower relative association strengths, except when indicated by ^§^ Abbreviations: *N*, number; *SD*, standard deviation. *The terms "Higher" and "Lower" pertain to the descriptive comparison of the risk of developing psoriatic arthritis, as indicated in **Supplemental Table 7**; e.g., the risk of PsA is 7.486% in psoriasis vulgaris and 1.198% for PPP. Hence, for the comparison of the two, psoriasis is labeled as “higher” and PPP as “lower”. Data retrieval was performed 10 months after the other analyses. Therefore, the sample size increased.

| **Diseases** | | **Higher descriptive risk** | | | | **Lower descriptive risk** | | | |  |  |  |
| --- | --- | --- | --- | --- | --- | --- | --- | --- | --- | --- | --- | --- |
| **Higher descriptive risk** | **Lower deskriptive risk** | ***N* of eligible participants** | ***N* of outcomes** | **Risk, %** | ***Median follow-up (days)*** | ***N* of eligible participants** | ***N* of outcomes** | **Risk, %** | ***Median follow-up (days)*** | **Risk difference (95% CI), %** | **HR**  **(95% CI)** | ***P* value** |
| Psoriasis Vulgaris | Pustulosis Palmoplantaris | 7,674 | 572 | 7.454 | 838 | 8,742 | 182 | 2.082 | 864.5 | 5.372  (4.712,6.031) | 3.825  (3.237,4.52) | < 0.0001 |
| Psoriasis Vulgaris | Generalized Pustular Psoriasis | 1,379 | 77 | 5.584 | 785 | 1,448 | 54 | 3.729 | 1,064 | 1.854  (0.298,3.41) | 1.872  (1.313,2.669) | 0.0004 |
| Generalized Pustular Psoriasis | Pustulosis Palmoplantaris | 1,407 | 54 | 3.838 | 1,019.5 | 1,482 | 28 | 1.889 | 855 | 1.949  (0.729,3.169% | 1.751  (1.104,2.777) | 0.0159 |

**Table 12.** Comparative risk of psoriatic arthritis development amongst varied psoriasis manifestations for EHRs indicating female sex. Abbreviations: *N*, number; *SD*, standard deviation. This analysis was performed 10 months after the other analyses. Therefore, the sample size increased.

| **Diseases Compared** | Psoriasis Vulgaris | Pustulosis Palmoplantaris | Psoriasis Vulgaris | Generalized Pustular Psoriasis | Generalized Pustular Psoriasis | Pustulosis Palmoplantaris |
| --- | --- | --- | --- | --- | --- | --- |
| ***Descriptive risk difference*** | ***Higher**** | ***Lower**** | ***Higher**** | ***Lower**** | ***Higher**** | ***Lower**** |
| ***N*** | 2,702 | 2,702 | 972 | 972 | 973 | 973 |
| **Age, years**  **Mean ± SD** | 50 ± 22.7 | 49.7 ± 23.2 | 37.1 ± 26.3 | 36.9 ± 26.6 | 36.5 ± 27 | 36.4 ± 27.3 |
| **Sex, female (%)** | 0 | 0 | 0 | 0 | 0 | 0 |
| **Ethnicity, white (%)** | 70.54 | 70.54 | 64.095 | 65.844 | 63.72 | 62.076 |

**Table 13.** Demographics of the study population in relative association analysis in the US Collaborative Network for EHRs indicating male sex. After propensity-matching for the shown variables, no significant differences were observed among all groups with the higher and lower relative association strengths, except when indicated. Abbreviations: *N*, number; *SD*, standard deviation. *The terms "Higher" and "Lower" pertain to the descriptive comparison of the risk of developing psoriatic arthritis, as indicated in **Supplemental Table 7**; e.g., the risk of PsA is 7.486% in psoriasis vulgaris and 1.198% for PPP. Hence, for the comparison of the two, psoriasis is labeled as “higher” and PPP as “lower”. Data retrieval was performed 10 months after the other analyses. Therefore, the sample size increased.

| **Diseases** | | **Higher descriptive risk** | | | | **Lower descriptive risk** | | | |  |  |  |
| --- | --- | --- | --- | --- | --- | --- | --- | --- | --- | --- | --- | --- |
| **Higher descriptive risk** | **Lower deskriptive risk** | ***N* of eligible participants** | ***N* of outcomes** | **Risk, %** | ***Median follow-up (days)*** | ***N* of eligible participants** | ***N* of outcomes** | **Risk, %** | ***Median follow-up (days)*** | **Risk difference (95% CI), %** | **HR**  **(95% CI)** | ***P* value** |
| Psoriasis Vulgaris | Pustulosis Palmoplantaris | 2,412 | 149 | 6.177 | 750 | 2,633 | 43 | 1.633 | 796 | 4.544  (3.468,5.62) | 4.024  (2.866,5.65) | < 0.0001 |
| Psoriasis Vulgaris | Generalized Pustular Psoriasis | 900 | 35 | 3.889 | 735 | 929 | 26 | 2.799 | 1,215 | 1.09  (-0.559,2.74) | 1.918  (1.14,3.225) | 0.0126 |
| Generalized Pustular Psoriasis | Pustulosis Palmoplantaris | 930 | 26 | 2.796 | 1,262 | 958 | 10 | 1.044 | 798 | 1.752  (0.512,2.992) | 2.488  (1.157,5.349) | 0.0158 |

**Table 14.** Comparative risk of psoriatic arthritis development amongst varied psoriasis manifestations for EHRs indicating male sex. Abbreviations: *N*, number; *SD*, standard deviation. This analysis was performed 10 months after the other analyses. Therefore, the sample size increased.

| **Characteristics** | **Psoriasis Vulgaris** | | | | **Pustulosis Palmoplantaris** | | | | **Generalized Pustular Psoriasis** | | | |
| --- | --- | --- | --- | --- | --- | --- | --- | --- | --- | --- | --- | --- |
|  | **Black or African American** | | **White** | | **Black or African American** | | **White** | | **Black or African American** | | **White** | |
|  | ***Cases*** | ***Controls*** | ***Cases*** | ***Controls*** | ***Cases*** | ***Controls*** | ***Cases*** | ***Controls*** | ***Cases*** | ***Controls*** | ***Cases*** | ***Controls*** |
| ***N*** | 2,121 | 2,121 | 23,043 | 23,043 | 971 | 971 | 5,634 | 5,634 | 486 | 486 | 1,432 | 1,432 |
| **Age, years**  **Mean ± SD** | 47.6 ± 18.8 | 47.6 ± 18.8 | 50.4 ± 18.8 | 50.4 ± 18.8 | 40.3 ± 26.2 | 40.3 ± 26.2 | 58.7 ± 18.5 | 58.7 ± 18.5 | 27.9 ± 25.4 | 27.9 ± 25.4 | 41.3 ± 26 | 41.3 ±  26 |
| **Sex, female (%)** | 57.0 | 57.0 | 52.7 | 52.7 | 64.8 | 64.8 | 78.7 | 78.7 | 60.7 | 60.7 | 56.1 | 56.1 |
| **Ethnicity, white (%)** | 0 | 0 | 100 | 100 | 0 | 0 | 100 | 100 | 0 | 0 | 100 | 100 |

**Supplemental Table 15.** Demographics of the study population in subgroup analysis for ethnicity. After propensity-matching for the shown variables, no significant differences were observed among all cases and controls, except for ethnicity. Abbreviations: *N*, number; *SD*, standard deviation.

|  | **Cases** | | | **Controls** | | |  |  |  |
| --- | --- | --- | --- | --- | --- | --- | --- | --- | --- |
| **Disease** | ***N* of eligible participants** | ***N* of outcomes** | **Risk, %** | ***N* of eligible participants** | ***N* of outcomes** | **Risk, %** | **Risk difference (95% CI), %** | **HR**  **(95% CI)** | ***P* value** |
| Psoriasis Vulgaris | 1,925 | 107 | 5.558 | 2,121 | 10* | 0.471 | 5.087  (4.023, 6.151) | 167.815  (23.194-1,214.217) | < 0.0001 |
| Pustulosis Palmoplantaris | 945 | 10* | 1.058 | 971 | 10* | 1.03 | 0.028  (-0.882, 0.939) | 16.117  (2.018-128.75) | 0.0005 |
| Generalized Pustular Psoriasis | 476 | 10* | 2.101 | 486 | 0 | 0 | 2.101  (0.812-3.389) | N/A | 0.0085 |

**Supplemental Table 16.** Risk of psoriatic arthritis development in varied psoriasis manifestations in Black or African American cases and controls. Abbreviations: *CI*, confidence interval; *HR,* hazard ratio; *N*, number, N/A, not applicable. *1-10 outcomes are by default displayed by 10 by TriNetX.

|  | **Cases** | | | **Controls** | | |  |  |  |
| --- | --- | --- | --- | --- | --- | --- | --- | --- | --- |
| **Disease** | ***N* of eligible participants** | ***N* of outcomes** | **Risk, %** | ***N* of eligible participants** | ***N* of outcomes** | **Risk, %** | **Risk difference (95% CI), %** | **HR**  **(95% CI)** | ***P* value** |
| Psoriasis Vulgaris | 19,867 | 1,441 | 7.253 | 22,998 | 41 | 0.178 | 7.075  (6.71, 7.44) | 59.36  (43.106-81.744) | < 0.0001 |
| Pustulosis Palmoplantaris | 5,464 | 71 | 1.299 | 5,628 | 13 | 0.231 | 1.068  (0.743, 1.394) | 8.004  (4.327-14.806) | < 0.0001 |
| Generalized Pustular Psoriasis | 1,346 | 39 | 2.897 | 1,431 | 10* | 0.699 | 2.199  (1.204, 3.193) | 20.658    (4.985-85.608) | < 0.0001 |

**Supplemental Table 17.** Risk of psoriatic arthritis development in varied psoriasis manifestations in White cases and controls. Abbreviations: *CI*, confidence interval; *HR,* hazard ratio; *N*, number.

| **Characteristics** | **Psoriasis Vulgaris** | |
| --- | --- | --- |
|  | **White** | **Black or African American** |
| ***Relative Assoc. Strength*** | ***Higher**** | ***Lower**** |
| ***N*** | 2,144 | 2,144 |
| **Age, years**  **Mean ± SD** | 47.4 ± 18.9 | 47.4 ± 18.9 |
| **Sex, female (%)** | 57.2 | 57.2 |
| **Ethnicity, white (%)** | 100 | 0 |

**Supplemental Table 18.** Demographics of the study population in subgroup relative association analysis (ethnicity). After propensity-matching for the shown variables, no significant differences were observed among all groups with the higher and lower relative association strengths, except for ethnicity. Abbreviations: *N*, number; *SD*, standard deviation. *The terms "Higher" and "Lower" pertain to the descriptive comparison of the risk of developing psoriatic arthritis, as indicated in **Supplemental** **Tables 14-15**.

| **Subgroups** | | **Higher Assoc. Strength** | | | **Lower Assoc. Strength** | | |  |  |  |
| --- | --- | --- | --- | --- | --- | --- | --- | --- | --- | --- |
| **Higher Assoc. Strength** | **Lower Assoc. Strength** | ***N* of eligible participants** | ***N* of outcomes** | **Risk, %** | ***N* of eligible participants** | ***N* of outcomes** | **Risk, %** | **Risk difference (95% CI), %** | **HR**  **(95% CI)** | ***P* value** |
| White | Black or African American | 1,851 | 133 | 7.185 | 1,946 | 107 | 5.498 | 1.687  (0.134, 3.239) | 1.338  (1.037- 1.726) | 0.0244 |

**Supplemental Table 19.** Risk of psoriatic arthritis development in Black or African American compared to White psoriasis vulgaris patients. Abbreviations: *CI*, confidence interval; *HR,* hazard ratio; *N*, number; *N/A*, not applicable.

| **Diseases Compared** | Psoriasis Vulgaris | Pustulosis Palmoplantaris | Psoriasis Vulgaris | Generalized Pustular Psoriasis | Generalized Pustular Psoriasis | Pustulosis Palmoplantaris |
| --- | --- | --- | --- | --- | --- | --- |
| ***Descriptive risk difference*** | ***Higher**** | ***Lower**** | ***Higher**** | ***Lower**** | ***Higher**** | ***Lower**** |
| ***N*** | 1,086 | 1,086 | 426 | 426 | 428 | 428 |
| **Age, years**  **Mean ± SD** | 47.3 ± 19.5 | 45.5 ± 22.9 | 34.3 ± 24.1^§^ | 33.5 ± 25^§^ | 32.8 ± 25.6^§^ | 32.9 ± 25.5^§^ |
| **Sex, female (%)** | 72.928 | 66.759 | 69.014 | 61.268 | 63.551^§^ | 64.019^§^ |
| **Ethnicity, white (%)** | 0 | 0 | 0 | 0 | 0 | 0 |

**Table 20.** Demographics of the study population in relative association analysis in the US Collaborative Network for EHRs indicating Black or African American ethnicity. After propensity-matching for the shown variables, no significant differences were observed among all groups with the higher and lower relative association strengths, except when indicated by ^§^. Abbreviations: *N*, number; *SD*, standard deviation. *The terms "Higher" and "Lower" pertain to the descriptive comparison of the risk of developing psoriatic arthritis, as indicated in **Supplemental Table 7**; e.g., the risk of PsA is 7.486% in psoriasis vulgaris and 1.198% for PPP. Hence, for the comparison of the two, psoriasis is labeled as “higher” and PPP as “lower”. Data retrieval was performed 10 months after the other analyses. Therefore, the sample size increased.

| **Diseases** | | **Higher descriptive risk** | | | | **Lower descriptive risk** | | | |  |  |  |
| --- | --- | --- | --- | --- | --- | --- | --- | --- | --- | --- | --- | --- |
| **Higher descriptive risk** | **Lower deskriptive risk** | ***N* of eligible participants** | ***N* of outcomes** | **Risk, %** | ***Median follow-up (days)*** | ***N* of eligible participants** | ***N* of outcomes** | **Risk, %** | ***Median follow-up (days)*** | **Risk difference (95% CI), %** | **HR**  **(95% CI)** | ***P* value** |
| Psoriasis Vulgaris | Pustulosis Palmoplantaris | 977 | 56 | 5.732 | 834 | 1,060 | 19 | 1.792 | 938 | 3.939  (2.277,5.601) | 3.539  (2.101,5.961) | < 0.0001 |
| Psoriasis Vulgaris | Generalized Pustular Psoriasis | 397 | 19 | 4.786 | 853.5 | 409 | 10 | 2.445 | 1,687 | 2.341  (-0.238,4.92) | 3.086  (1.361,6.998) | 0.0047 |
| Generalized Pustular Psoriasis | Pustulosis Palmoplantaris | 411 | 10 | 2.433 | 1,638.5 | 417 | 10 | 2.398 | 895.5 | 0.035  (-2.057,2.127) | 0.929  (0.355,2.428) | 0.8803 |

**Table 21.** Comparative risk of psoriatic arthritis development amongst varied psoriasis manifestations for EHRs indicating Black or African American ethnicity. Abbreviations: *N*, number; *SD*, standard deviation. This analysis was performed 10 months after the other analyses. Therefore, the sample size increased.

| **Diseases Compared** | Psoriasis Vulgaris | Pustulosis Palmoplantaris | Psoriasis Vulgaris | Generalized Pustular Psoriasis | Generalized Pustular Psoriasis | Pustulosis Palmoplantaris |
| --- | --- | --- | --- | --- | --- | --- |
| ***Descriptive risk difference*** | ***Higher**** | ***Lower**** | ***Higher**** | ***Lower**** | ***Higher**** | ***Lower**** |
| ***N*** | 1,165 | 1,165 | 1,652 | 1,652 | 1,595 | 1,595 |
| **Age, years**  **Mean ± SD** | 44.9 ± 21.5^§^ | 42.5 ± 24.8^§^ | 44.4 ± 24 | 44.2 ± 24.3 | 45.5 ± 23.8 | 46.1 ± 22.9 |
| **Sex, female (%)** | 73.734^§^ | 73.734^§^ | 62.167 | 61.138 | 64.451^§^ | 60.94^§^ |
| **Ethnicity, white (%)** | 100 | 100 | 100 | 100 | 100 | 100 |

**Table 22.** Demographics of the study population in relative association analysis in the US Collaborative Network for EHRs indicating White ethnicity. After propensity-matching for the shown variables, no significant differences were observed among all groups with the higher and lower relative association strengths, except when indicated by ^§^. Abbreviations: *N*, number; *SD*, standard deviation. *The terms "Higher" and "Lower" pertain to the descriptive comparison of the risk of developing psoriatic arthritis, as indicated in **Supplemental Table 7**; e.g., the risk of PsA is 7.486% in psoriasis vulgaris and 1.198% for PPP. Hence, for the comparison of the two, psoriasis is labeled as “higher” and PPP as “lower”. Data retrieval was performed 10 months after the other analyses. Therefore, the sample size increased.

| **Diseases** | | **Higher descriptive risk** | | | | **Lower descriptive risk** | | | |  |  |  |
| --- | --- | --- | --- | --- | --- | --- | --- | --- | --- | --- | --- | --- |
| **Higher descriptive risk** | **Lower deskriptive risk** | ***N* of eligible participants** | ***N* of outcomes** | **Risk, %** | ***Median follow-up (days)*** | ***N* of eligible participants** | ***N* of outcomes** | **Risk, %** | ***Median follow-up (days)*** | **Risk difference (95% CI), %** | **HR**  **(95% CI)** | ***P* value** |
| Psoriasis Vulgaris | Pustulosis Palmoplantaris | 1,009 | 72 | 7.136 | 848 | 1,139 | 19 | 1.668 | 950 | 5.468 | 4.913  (2.961,8.151) | < 0.0001 |
| Psoriasis Vulgaris | Generalized Pustular Psoriasis | 1,438 | 88 | 6.12 | 835.5 | 1,526 | 59 | 3.866 | 1,114 | 2.253  (0.682,3.825) | 1.996  (1.422,2.8) | < 0.0001 |
| Generalized Pustular Psoriasis | Pustulosis Palmoplantaris | 1,469 | 60 | 4.084 | 1,071 | 1,546 | 33 | 2.135 | 846 | 1.95  (0.707,3.192) | 1.612  (1.048,2.48) | 0.0284 |

**Table 23.** Comparative risk of psoriatic arthritis development amongst varied psoriasis manifestations for EHRs indicating White ethnicity. Abbreviations: *N*, number; *SD*, standard deviation. This analysis was performed 10 months after the other analyses. Therefore, the sample size increased.
